# Editorial: Sex Hormone Fluctuations Across the Female Lifespan: Mechanisms of Action on Brain Structure, Function, and Behavior

**DOI:** 10.3389/fnbeh.2022.964740

**Published:** 2022-07-05

**Authors:** Stephanie V. Koebele, Alexandra Ycaza Herrera, Caitlin M. Taylor, Claudia Barth, Jaclyn M. Schwarz

**Affiliations:** ^1^Department of Psychology, Arizona State University, Tempe, AZ, United States; ^2^Arizona Alzheimer's Consortium, Phoenix, AZ, United States; ^3^Leonard Davis School of Gerontology, University of Southern California, Los Angeles, CA, United States; ^4^Department of Psychological and Brain Sciences, University of California, Santa Barbara, Santa Barbara, CA, United States; ^5^Department of Psychiatric Research, Diakonhjemmet Hospital, Oslo, Norway; ^6^Norwegian Centre for Mental Disorders Research, Institute of Clinical Medicine, University of Oslo, Oslo, Norway; ^7^Department of Psychological and Brain Sciences, University of Delaware, Newark, DE, United States

**Keywords:** estrogens, progestogens, androgens, menstrual cycle, menopause, women's health, oral contraceptive, sex hormones

Medical and scientific literature, from preclinical animal models to clinical trials, largely excludes females (Woitowich et al., 2020). The justifications for exclusion are manifold, with the cyclic nature of hormones across the female lifespan targeted as a source of irremediable confound (Shansky, 2019; Rechlin et al., 2022). This dogma has led to an overreliance on data from male participants (Geller et al., 2018). As a result, significant disparities exist in our understanding of the female brain and body across the lifespan (Taylor et al., 2020; Shansky and Murphy, 2021). This perpetuates inequities, such that women frequently experience delays in receiving a diagnosis, which results in inferior healthcare (Vlassoff, 2007; Westergaard et al., 2019; Chinn et al., 2021). Important research over the past several decades has taught the field that sex hormones, including estrogens, androgens, and progesterone, have far-reaching effects beyond their classic reproductive functions. This Research Topic highlights the ways in which sex hormones exert broad systems-levels effects on mammalian biology, including the central nervous system. These effects are not static, but rather fluctuate across time scales ranging from diurnal to decades in both males and females. Major hormone transition periods, including puberty/adolescence, the menstrual cycle, pregnancy, the postpartum period, and the menopause transition impart unique effects on the female brain that can alter the trajectory of brain and cognitive aging, resulting in long lasting structural and functional brain changes (Koebele and Bimonte-Nelson, 2015). The contributions to this collection not only expand our understanding of the impact of sex hormones on the brain and behavior, but will also allow researchers and healthcare professionals to better serve individuals who have an incredible depth and breadth of diverse hormone-related experiences and exposures across the lifespan.

With regard to endogenous hormone fluctuations across the menstrual cycle, Diekhof et al. provide an intriguing report on the influence of variations in 17β-estradiol levels and the COMT-Val158Met genotype on decision-making across the menstrual cycle. Xu et al. also present novel findings demonstrating that varied workload demands modulate menstrual cycle effects on attentional processes. These studies help reveal the complexity and nuance of sex hormones' influence on behavior.

Preclinical and clinical perspectives have elucidated exogenous hormone effects on the brain and behavior, including hormone-containing contraceptives and menopausal hormone therapies. Despite a majority of women having exposure to exogenous hormones during their lifetime (Centers for Disease Control Prevention, 2019), this is a profoundly understudied research area. In their topic contribution, Beltz et al. explore biopsychosocial influences on spatial skills, including those of oral contraceptive use and gender self-concept on a mental rotation task, expanding our understanding of factors contributing to performance variability often reported from neuropsychological tasks. Kimmig et al. demonstrate that oral contraceptives do not impair emotion recognition, and that progesterone levels were associated with cycle-dependent differences in negativity bias in naturally cycling women during an emotion recognition task. Mentin-Henry et al. also provide novel insights into emotional processing using resting state functional magnetic resonance imaging by reporting both sex and oral contraceptive use alter brain and behavioral outcomes for their emotion recognition task; interestingly, the androgenicity of oral contraceptives significantly impacted results. In a complimentary fashion, Koebele et al. showed that variations in hormone therapy formulation differentially impact working memory, anxiety-like and depressive-like behavior outcomes in a preclinical rat model of transitional menopause. Together, these findings underscore the need to take an individualized approach to exogenous hormone therapies rather than the one-size-fits-all approach that is commonplace today.

Sex hormones modulate all body systems including the brain, which is substantiated by clear sex differences in the incidence of a number of psychiatric, autoimmune, and neurological diseases (Barth et al., 2015; Mauvais-Jarvis et al., 2020). Moreover, mounting evidence suggests that these systemic influences interact. For instance, Engler-Chiurazzi et al. share an in-depth review exploring the convergence of estrogen effects on immune function and affect from a lifespan perspective, advancing the field's knowledge of endocrine–immune interactions in the context of mental health. Furthermore, the critical window hypothesis of hormone loss and cognitive impairment has driven the field's exploration of cognitive changes during aging in recent decades (Maki, 2013). Rodríguez-Landa contributes an insightful commentary on the importance of hormone intervention timing when designing preclinical experiments to investigate affective behavior. This discussion will aid our understanding and interpretation of the time course of hormone and brain changes following surgical menopause intervention.

It is also imperative to illuminate the neurobiological mechanisms underpinning cognitive–behavioral changes across multiple time scales. Beamish and Frick propose a novel mechanism through which estrogen exerts its effects on hippocampal-dependent learning and memory by exploring the role of the ubiquitin proteasome system in synapse remodeling in both sexes. Jiménez-Balado et al. provide new insights into neural mechanisms underlying episodic memory by utilizing magnetic resonance spectroscopy to demonstrate that the relationship between lower hippocampal γ-aminobutyric acid (GABA) concentrations and poorer episodic memory is driven by sex, not apolipoprotein E (ApoE) ε4 genotype, as the effect was only observed in older community-dwelling women. Gilfarb and Leuner also contribute a thoughtful review summarizing the field's current understanding of cognitive–behavioral effects related to changes in the GABAergic system across major hormonal transition periods from puberty to menopause. Understanding the mechanisms underlying cognitive–behavioral effects of sex hormones is key to ultimately enhancing precision medicine approaches and care at every life stage.

Collectively, investigating the female brain and body across the lifespan with intention not only provides opportunities for advancement in the field of women's health, but also permits us to discover more about the broader human experience through inclusive research practices. Elucidating mechanisms of action of sex hormones on brain structure, function, and behavior allows us to acknowledge the value of these molecules at each life stage. Despite the decades-long axiom that female hormone fluctuations introduce unwanted variability in research, we must recognize that inherent hormone fluctuations across the female lifespan are not pathological or inconsequential, but rather an intrinsic and integral property of the female experience that allows for lifelong neural plasticity and resilience. By emphasizing sex and gender as key biological variables in research inquiries, we will gain knowledge of the human experience as well as improve healthcare and quality of life for women and gender diverse individuals across all life stages. We also hope that this growing awareness of the female experience will provide support for women to have equal and equitable access to essential healthcare across the lifespan.

## Author Contributions

SK: conceptualization, writing-original draft, and writing—review and editing. AYH, CT, CB, and JS: conceptualization and writing—review and editing. All authors contributed to the article and approved the submitted version.

## Conflict of Interest

The authors declare that the research was conducted in the absence of any commercial or financial relationships that could be construed as a potential conflict of interest.

## Publisher's Note

All claims expressed in this article are solely those of the authors and do not necessarily represent those of their affiliated organizations, or those of the publisher, the editors and the reviewers. Any product that may be evaluated in this article, or claim that may be made by its manufacturer, is not guaranteed or endorsed by the publisher.
